# Unique properties of Drosophila spermatocyte primary cilia

**DOI:** 10.1242/bio.20135355

**Published:** 2013-09-11

**Authors:** Maria Giovanna Riparbelli, Oscar A. Cabrera, Giuliano Callaini, Timothy L. Megraw

**Affiliations:** 1 Department of Evolutionary Biology, University of Siena, Via Aldo Moro 4, I-53100 Siena, Italy; 2 Department of Biomedical Sciences, Florida State University College of Medicine, Tallahassee, FL 32306-4300, USA

**Keywords:** Drosophila, Centriole, Centrosome, Cilia, Spermatocyte, Transition zone

## Abstract

The primary cilium is an essential organelle required for animal development and adult homeostasis that is found on most animal cells. The primary cilium contains a microtubule-based axoneme cytoskeleton that typically grows from the mother centriole in G0/G1 phase of the cell cycle as a membrane-bound compartment that protrudes from the cell surface. A unique system of bidirectional transport, intraflagellar transport (IFT), maintains the structure and function of cilia. While the axoneme is dynamic, growing and shrinking at its tip, at the same time it is very stable to the effects of microtubule-targeting drugs. The primary cilia found on Drosophila spermatocytes diverge from the general rules of primary cilium biology in several respects. Among these unique attributes, spermatocyte cilia assemble from all four centrioles in an IFT-independent manner in G2 phase, and persist continuously through two cell divisions. Here, we show that Drosophila spermatocyte primary cilia are extremely sensitive to microtubule-targeting drugs, unlike their mammalian counterparts. Spermatocyte cilia and their axonemes fail to assemble or be maintained upon nocodazole treatment, while centriole replication appears unperturbed. On the other hand, paclitaxel (Taxol), a microtubule-stabilizing drug, disrupted transition zone assembly and anchoring to the plasma membrane while causing spermatocyte primary cilia to grow extensively long during the assembly/elongation phase, but did not overtly affect the centrioles. However, once assembled to their mature length, spermatocyte cilia appeared unaffected by Taxol. The effects of these drugs on axoneme dynamics further demonstrate that spermatocyte primary cilia are endowed with unique assembly properties.

## Introduction

Primary cilia are essential organelles for animal development and human disease. Cilia are assembled from plasma membrane-tethered basal bodies that project their microtubule-based axonemes from the cell surface surrounded by a membranous sheath. The cilium is the cell's “antenna”: a specialized membrane domain enriched in specific receptors that detect extracellular signals. Primary cilia are involved in a variety of functions from cell fate determination during embryogenesis, to tissue maintenance and homeostasis (Goetz and Anderson, 2010; Hildebrandt et al., 2011; Berbari et al., 2009; Bettencourt-Dias et al., 2011; Nigg and Raff, 2009; Azimzadeh and Marshall, 2010). Defects in cilia assembly, structure or function lead to disorders of heterogeneous severity collectively referred to as ciliopathies. Therefore, a deeper understanding of cilium mechanics is essential for developing treatments for ciliopathies.

The formation of primary cilia requires the migration of centrioles from the inner cytoplasm to the cell surface and their docking with the plasma membrane, a process that requires centriole conversion to functional basal bodies capable of ciliary axoneme assembly. The assembly and elongation of cilia and flagella depend on a conserved intraflagellar transport (IFT) process powered by IFT-associated motors (Rosenbaum and Witman, 2002). IFT is required for assembly of the chordotonal sensory cilia in the fruit fly Drosophila melanogaster, where mutations in the IFT-A components like rempA (Lee et al., 2008), IFT-B components like NompB (Sarpal et al., 2003) and the heterotrimeric kinesin-2 Klp64D (Han et al., 2003) impair cilia assembly, resulting in loss of sensory function. In contrast, mutant males for these genes had apparently normal sperm axonemes (Han et al., 2003; Sarpal et al., 2003). It is therefore puzzling that the remarkably long D. melanogaster sperm axoneme, 1.8 mm in length, does not require the IFT transport machinery to assemble, whereas most primary cilia, including the 200-times shorter Drosophila neuronal cilia requires IFT for assembly. A possible explanation for this discrepancy could be the peculiar mode of sperm axoneme assembly in Drosophila that occurs within the cytoplasm, thus affording diffusible access of the assembly components to the growing axoneme in spermatids (Tokuyasu, 1975; Witman, 2003). However, the distal-most 5–10-µm tip of the axoneme, originally referred to as the “cilium” (Tokuyasu, 1975), is surrounded by a continuous plasma membrane that makes addition of distal axoneme components by a diffusion-dependent mechanism a conceptually less-favorable model. Thus, why sperm flagellum assembly is not reliant on conventional IFT remains a conundrum.

Early in spermatogenesis, Drosophila spermatocytes assemble primary cilia that persist through two meiotic divisions (Riparbelli et al., 2012) and appear to be precursors of the spermatid flagellum (Tokuyasu, 1975). This contrasts with vertebrate cells where mitosis does not proceed without cilium resorption (Kim and Tsiokas, 2011; Kim et al., 2011; Kobayashi and Dynlacht, 2011; Pan and Snell, 2007; Pearson et al., 2007; Plotnikova et al., 2009; Quarmby and Parker, 2005; Rieder et al., 1979; Santos and Reiter, 2008; Seeley and Nachury, 2010). Since spermatocyte primary cilia are surrounded by a continuous plasma membrane and otherwise appear like conventional yet short cilia, their assembly and elongation are predicted to require IFT. However, mutations in IFT components do not affect assembly of the spermatocyte primary cilium (Riparbelli et al., 2012). Thus, the dynamics of these structures appear to be IFT-independent, suggesting alternative mechanisms of axoneme elongation that offer a unique model to investigate IFT-independent mechanisms of primary cilium assembly.

Disassembly of cytoplasmic microtubules impairs cilia formation in cultured vertebrate cells (Stubblefield and Brinkley, 1966; Jensen et al., 1987; Boisvieux-Ulrich et al., 1989a). Moreover, Taxol, which promotes polymerization and stabilization of microtubules, disturbed ciliogenesis in quail oviduct cells (Boisvieux-Ulrich et al., 1989b) and inhibited cilia elongation in mammalian cultured cells (Sharma et al., 2011). Because the IFT particles carry the components for axoneme assembly to the tip of the growing cilium from cytoplasmic pools via motor proteins, the concentration of soluble cytoplasmic tubulin affects ciliary dynamics (Sharma et al., 2011). Low concentrations of microtubule disrupting agents resulted in the elongation of the primary cilia (Kim et al., 2010; Bershteyn et al., 2010) due to a consequent increase of soluble tubulin (Sharma et al., 2011). These data implicate a direct correlation between the modulation of cilia dynamics and the assembly status of the cytoplasmic microtubule network and its impact on the availability of tubulin.

Because the primary cilia in Drosophila spermatocytes assemble independent of IFT (Riparbelli et al., 2012), this prompted us to investigate the role of the microtubule cytoskeleton on the dynamics of these cilia by examining the assembly and elongation of these structures under conditions where the polymerization of the cytoplasmic microtubules has been altered by pharmacological interventions. Here we show that, unlike primary cilia in mammalian cells, the axoneme of Drosophila spermatocyte cilia are directly impacted by drugs that target microtubule polymerization. In the presence of nocodazole, a microtubule destabilizer, cilia axonemes failed to assemble, and with Taxol, a stabilizer, the axonemes grew unusually long. In addition, the effects of Taxol on cilium assembly were more complex, and did not affect the axoneme once cilium assembly was complete.

## Results and Discussion

Gametogenesis in Drosophila males starts with asymmetric division of the germline stem cells. Each division produces a renewed stem cell and a primary spermatogonium. Primary spermatogonia then undergo four rounds of mitosis with incomplete cytokinesis, forming cysts of 16 interconnected secondary spermatogonia that then begin meiosis as primary spermatocytes. Spermatogonial centrioles duplicate each cell division cycle and the young primary spermatocytes inherit a centriole pair that replicate at the beginning of the G2 phase (A. D. Tates, Cytodifferentiation during spermatogenesis in Drosophila melanogaster: an electron microscopy study, PhD thesis, Rijksunivrsiteit de Leiden, Netherlands, 1971). The two centriole pairs, which reside near the nucleus at the beginning of the first meiotic prophase, then migrate toward the cell periphery and dock with the plasma membrane as the primary spermatocytes grow. At the plasma membrane all four docked centrioles function as basal bodies and assemble axonemes of atypical cilia that persist through two meiotic divisions until the onset of spermiogenesis (Riparbelli et al., 2012).

The product of the *uncoordinated* (*unc*) gene, which is required for ciliogenesis and mechanosensation in Drosophila, localizes to the distal tip of the centriole and is a marker for conversion of the centriole to a basal body (Baker et al., 2004). Unc localization to the distal tips of centrioles coincides with basal body docking at the spermatocyte plasma membrane (Riparbelli et al., 2012), and is thought to reside in the transition zone (Ma and Jarman, 2011; Enjolras et al., 2012), a compartment that marks the transition from basal body to cilium (Reiter et al., 2012; Szymanska and Johnson, 2012). Thus, Unc recruitment to centrioles is a marker for cilium assembly. However, the localization of Unc to the transition zone has not been shown definitively by immuno-EM, so here we will refer to the compartment that contains the enriched localization of Unc as the distal centriole compartment (DCC).

### Centriole duplication during male gametogenesis is not blocked by nocodazole

While spindle checkpoints are present during male gametogenesis of Drosophila (Rebollo and González, 2000), the depolymerization of microtubules by nocodazole or their stabilization upon Taxol treatment does not lead to a metaphase arrest, as occurs in some somatic cells, but rather a delay of about 16 min in anaphase onset. Therefore, some aspects of meiosis continue, although in an irregular manner.

**Fig. 1.**
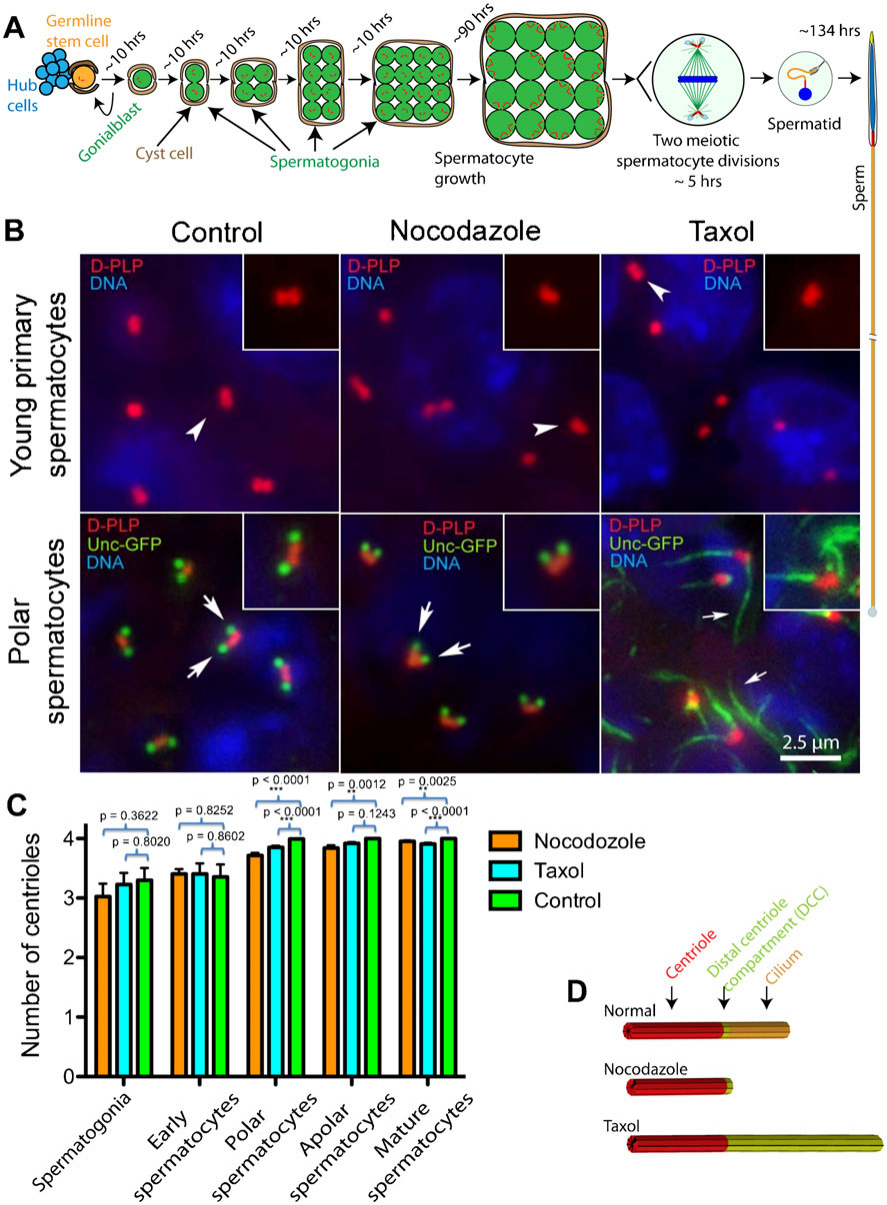
Nocodazole and Taxol do not impede centriole duplication. (A) Spermatogenesis in Drosophila. The timing of divisions or developmental phases is indicated. Cells and organelles are not drawn to scale, and a single meiotic spermatocyte and spermatid rather than the entire cyst is shown for simplicity. (B) Microtubule depolymerization or stabilization does not affect the duplication of centrioles, labelled by anti-D-PLP staining (arrowheads) in young primary spermatocytes. Unc-GFP localization at the distal ends of centrioles (arrows) begins at the polar spermatocyte stage. Unc-GFP localization is not altered in polar spermatocytes following microtubule depolymerization with nocodazole. In contrast, following Taxol treatment, Unc-GFP is localized along filamentous structures extending from the distal centrioles (small arrows). (C) Quantification of centriole numbers at different stages of spermatogenesis following 24 hr nocodazole or Taxol treatment. Error bars represent SEM. (D) Schematic of the centriole–cilium complex in normal, nocodazole- and Taxol-treated spermatocytes. The localization of Unc defines the DCC (light green) at normal cilia. Scale bar: 2.5 µm.

To determine whether centriole replication and maturation to basal bodies in young primary spermatocytes requires intact or dynamic microtubules, we examined centriole replication and their transition to basal bodies in testes that were cultured with 10 µM nocodazole or 5 µM Taxol for 24 hrs. Spermatogonia have a cell cycle length of about 10 hours (Lindsley and Tokuyasu, 1980), so at least one round of duplication should have occurred prior to the visualization of early primary spermatocytes (Fig. 1A). To determine whether these treatments affected centriole replication or maturation to basal bodies, we examined centrioles and their transition to basal bodies using D-PLP, a centriolar protein, and Unc-GFP as markers, respectively. D-PLP localization appeared normal in young primary spermatocytes treated with nocodazole or Taxol for 24 hrs (Fig. 1B), and there was no significant difference in centriole numbers (Fig. 1C). These data indicate that centriole duplication was not impeded by microtubule disruption. The efficacy of drug treatments was evident by the lack of visible microtubules following 10 µM nocodazole treatment or by the presence of thick microtubule arrays by 5 µM Taxol (not shown). Together, these data indicate that centrioles duplicated successfully during spermatogonial divisions and in early spermatocytes, regardless of drug treatment.

In later spermatocytes, at a stage when no centriole replication was expected to occur in the preceding 24 hrs of treatment, there was a slight but statistically significant drop in centriole numbers in both nocodazole- and Taxol-treated cells (Fig. 1C). This suggests that centrioles are lost or disassembled at a low frequency under these conditions.

It has been reported that nocodazole and colchicine block the assembly of centrioles/basal bodies while not causing disassembly of existing ones (Boisvieux-Ulrich et al., 1989a; Jensen et al., 1987; Balczon et al., 1999; Le Clech, 2008). In contrast, here we show that centriole duplication during spermatogonial and spermatocyte divisions are not inhibited in Drosophila by high concentration (10 µM) nocodazole treatment, whereas the ciliary axoneme disassembles or fails to assemble (see below).

Unc localized to the DCC at the polar spermatocyte stage in nocodazole-treated cells, as normally occurs in control cells (Fig. 1B,D), indicating that centrioles matured to basal bodies in nocodazole-treated cells. On the other hand, in Taxol-treated spermatocytes, Unc localized to long filaments extending from the distal tip of each centriole, suggesting that Taxol promotes the assembly of unusually long cilia (Fig. 1B,D). Next, we examined the effects of nocodazole and Taxol on the assembly of spermatocyte cilia.

#### Assembly of spermatocyte primary cilia requires intact microtubules

**Fig. 2.**
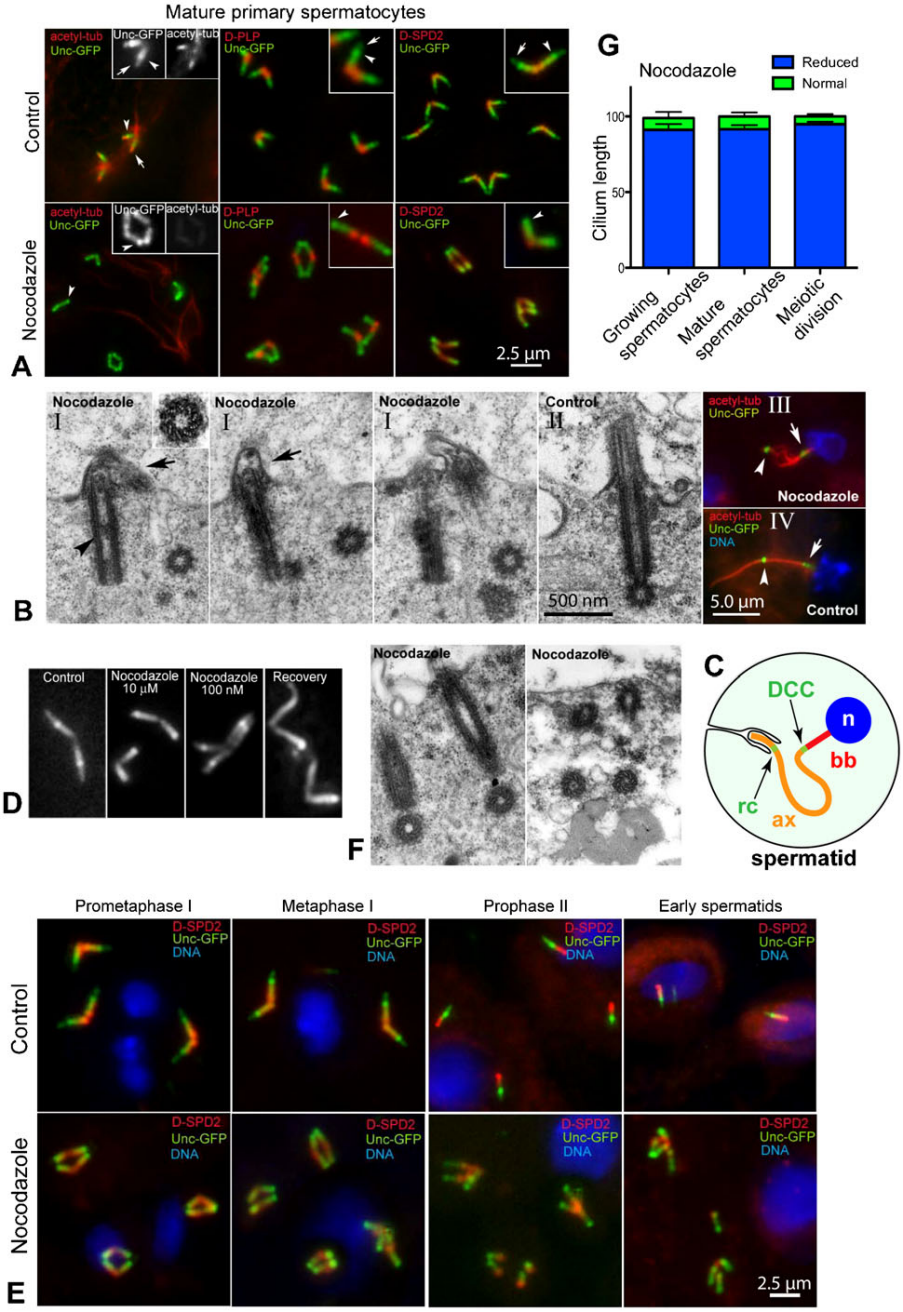
Spermatocyte primary cilia do not assemble in the presence of nocodazole. (A) Unc-GFP localizes to a bright dot just distal to the centriole (arrowhead), and a weaker signal for Unc-GFP is detected distal to that along the axoneme and also on the centriole. Acetylated tubulin is detected in the ciliary axoneme (arrow) distal to the compartment that is labelled prominently with Unc-GFP at the distal centriole. Staining of centrioles with antibodies against D-PLP or D-SPD2 show the localization pattern of Unc-GFP relative to the centriole. Nocodazole treatment disrupts the axoneme signal for acetylated tubulin and Unc-GFP, but does not disrupt the centrioles or the Unc-GFP signal at the centriole. (B) (I) Consecutive serial sections showing the lack of a ciliary axoneme in nocodazole-treated spermatocytes and the presence of irregular membrane bulging above the centriole (arrows); the lumen of the centriole contains electron-dense material (arrowhead). Inset shows a cross section of a centriole. (II) Centriole and cilium in control primary spermatocytes. The axoneme of elongating spermatids is disorganized after nocodazole treatment (III) relative to control spermatids (IV); Unc-GFP is localized to the centriole (arrows) and to a distinct spot, the ring centriole (arrowheads), along the axoneme. (C) Cartoon of the early spermatid with a growing flagellum. Labels: rc, ring centriole; DCC, distal centriole compartment; bb, basal body; n, nucleus; ax, axoneme. (D) High concentrations of nocodazole (10 µM) disrupt both cytoplasmic microtubules and the ciliary structures, whereas lower concentrations (100 nM) led to cytoplasmic microtubule depolymerization (not shown) but leave the ciliary axoneme intact; recovery for 12 hours from nocodazole leads to the development of an abnormal cilium-like projection. (E) In control primary spermatocytes the centriole pairs separate during prophase, and the centrioles of each pair then separate during meiotic progression; by contrast, centriole pairs did not separate and individual centrioles seldom separated when treated with nocodazole. (F) Section of a nocodazole-treated primary spermatocyte showing two pairs of adjacent centrioles (see also panel B). (G) Quantification of cilium disruption scored in the indicated cell types based upon the presence of the Unc-GFP signal distal to the DCC. Error bars represent SEM. Scale bars: 2.5 µm (A,E), 500 nm (B) (II), 5 µm (B) (IV).

In late spermatocytes, during their extended growth phase (G2), Unc-GFP is localized prominently to a focus at the DCC, and is also localized weakly along the distal half of the centriole and along the cilium (Baker et al., 2004; Riparbelli et al., 2012) (Fig. 2A). This pattern was altered in nocodazole and Taxol treated cells. Co-staining for D-PLP or D-SPD2, which localize to the proximal end of centrioles and along the entire length of centrioles, respectively, shows that in nocodazole-treated spermatocytes Unc localizes to centrioles and prominently to the DCC, but the signal along the cilium is no longer detectable (Fig. 2A), indicating that nocodazole blocks axoneme microtubule assembly. To examine the axoneme more directly, we stained nocodazole-treated spermatocytes with anti-acetylated tubulin antibodies, which recognize the microtubules of ciliary axonemes but not the centrioles in spermatocytes (Piperno and Fuller, 1985; Riparbelli et al., 2012). Acetylated tubulin was undetectable in the axoneme following nocodazole treatment (Fig. 2A). Thus, nocodazole appears to block axoneme assembly of spermatocyte cilia.

To directly view the alteration of axoneme structures in nocodazole-treated spermatocytes, we examined ultrathin serial sections by transmission electron microscopy (TEM). The results confirmed that nocodazole-treated mature primary spermatocytes lacked a distinct ciliary axoneme (Fig. 2BI), which is clearly evident in control cilia (Fig. 2BII). While the axoneme was disrupted, the architecture of the centriole was unchanged and a distinct cartwheel was present (Fig. 2BI, inset). In contrast, the central tubule that is found within the lumen of the spermatocyte centriole (Riparbelli et al., 2012; Carvalho-Santos et al., 2012; Roque et al., 2012) was absent and in its place an electron-dense aggregate was frequently found (Fig. 2BI). The docked basal body produces a dome-shaped protrusion at the plasma membrane (Fig. 2BI). This plasma membrane protrusion appears similar to those seen at the early stages of cilium formation in young control spermatocytes (A. D. Tates, PhD thesis; Riparbelli et al., 2012). Together, these data indicate that centriole duplication, maturation to basal body, migration, and docking to the cell membrane occurs during nocodazole treatment, but the assembly of the ciliary axoneme is blocked.

A remaining question is whether the ciliary axoneme forms and then disassembles in nocodazole-treated cells. The protrusion of the plasma membrane at the distal end of the centrioles following nocodazole treatment (Fig. 2BI) suggests that an axoneme may initially form and then depolymerize, leaving a bulging plasma membrane after retracting. Nocodazole may therefore inhibit ciliary axoneme formation and elongation and may target the disassembly of the axonemal doublets in ciliary axonemes already formed before drug treatment. This scenario suggests that the axonemal doublet microtubules are less stable than centriolar triplet microtubules. By contrast, recovery from 10 µM nocodazole for 12 hours led to the formation of irregular cilia in about 30% of growing primary spermatocytes (Fig. 2D), suggesting that cilium assembly proceeds past a quality control point when microtubules are depolymerized.

In spermatids, a cilium, encased in plasma membrane that invaginates from the cell surface, remains as the axoneme grows (Tokuyasu, 1975). This odd configuration, where a cilium-like structure is separated from the basal body but connected by a growing axoneme, is marked by two separate foci of Unc (Fig. 2C). The structure at the base of the spermatid cilium, perhaps similar to a transition zone but lacks a centriole, has been given the name “ring centriole” (Phillips, 1970), even though there is no apparent basal body there. Unc appears to localize to the ring centriole in addition to the DCC in spermatids (Wei et al., 2008; Fabian and Brill, 2012). In addition to the effects on the spermatocyte cilium, nocodazole perturbed the organization of the flagellar axoneme in elongating spermatids, which appeared splayed (Fig. 2BIII) compared to control spermatids (Fig. 2BIV), and appears to disassemble the cilium distal to the ring centriole (Fig. 2BIV,C).

Treatment of spermatocytes with a 100-fold lower nocodazole concentration (0.1 µM) disrupted the cytoplasmic microtubules and inhibited centriole separation, similar to the effects with 10 µM nocodazole. However, in contrast to the disruption of spermatocyte cilia by 10 µM nocodazole, 0.1 µM nocodazole did not affect the length of ciliary axonemes and the pattern of Unc distribution on the centriole–cilium complex was similar to the controls (Fig. 2D).

Together, these data indicate that the centriole and the ciliary axoneme, two distinct but continuous microtubule based structures, have different sensitivities to nocodazole. This implies that something protects microtubules from nocodazole in the centriole, but not in its apical extension, the ciliary axoneme. The nature of these differences, whether they are posttranslational modifications, associated cofactors that impact microtubule stability, or other considerations are unclear.

It was previously shown that disassembly of cytoplasmic microtubules in Chinese hamster fibroblasts (Stubblefield and Brinkley, 1966), quail oviduct cells (Boisvieux-Ulrich et al., 1989a) and Ptk1 cells (Jensen et al., 1987) blocked primary cilia assembly. In agreement with these reports, we found that the organization of primary cilia in Drosophila spermatocytes was severely disrupted by nocodazole. It was also shown in vertebrate cells that microtubule depolymerization did not affect the organization of cilia that were assembled prior to drug treatment. This effect contrasts with our findings. Instead, we find that axoneme assembly or maintenance in primary and secondary spermatocyte cilia and in spermatid flagella were inhibited by nocodazole. Since we implemented these experiments on testes harvested from the mid-pupal stage, they consist of spermatocytes at various stages of ciliogenesis. We therefore conclude that nocodazole not only inhibits assembly of spermatocyte cilia, but also causes their disassembly.

In vertebrate cells, both the IFT and the cytosolic microtubule transport systems, owing to the action of microtubule-based motor proteins that move cargos along the microtubule network, are disrupted in the presence of high concentration of nocodazole, affecting assembly dynamics of the ciliary axoneme. Strikingly, low concentrations of microtubule depolymerizing drugs increased axoneme length in primary cilia (Sharma et al., 2011). This effect was attributed to an increase of the free tubulin pool available to IFT for ciliary axoneme growth. In contrast to those findings, we found that a low concentration of nocodazole (0.1 µM) resulted in significant depolymerization of the cytoplasmic microtubules but had no overt effect on the cilium assembly in Drosophila spermatocytes (Fig. 2D). This result suggests that cilium assembly in Drosophila spermatocytes and the mode of tubulin delivery is different from that of vertebrate cells, possibly because IFT is not required for cilium assembly in Drosophila testis (Han et al., 2003; Sarpal et al., 2003; Riparbelli et al., 2012).

#### Centrosome separation requires intact microtubules

Each primary spermatocyte has two pairs of “engaged” orthogonally-arranged centrioles, and each of the four centrioles organizes a cilium (Riparbelli et al., 2012). Centrioles elongate during the long G2 phase and the pairs move apart at prometaphase to reach the opposite sides of the cell, where they organize the poles of the metaphase spindle. During late anaphase of the first meiosis the centriole pair disengages and each centriole separates from each other (Fig. 2E). Thus, telophase spindle poles of primary spermatocytes normally display two separated centrioles. In the next division, meiosis II, the centrioles do not replicate and one centriole is found at each spindle pole (Fig. 2E). Finally, young spermatids at the onset of spermiogenesis inherit a single centriole that retains a remnant of the cilium (Fig. 2E).

We found that a prominent effect of nocodazole was a block in separation of centriole pairs, and a tendency of centriole pairs to aggregate at their distal tips, assuming a tip-to-tip configuration. This is visible by fluorescent staining (Fig. 2A,E) and by TEM (Fig. 2BI,F). The TEM images indicate that these distal associations occur between the small membrane protrusions (which also lack a normal axoneme, see below) (Fig. 2BI). Early spermatids that resulted from the failed division of nocodazole-treated primary or secondary spermatocytes had two or four adjacent centrioles, instead of the single centriole in a normal spermatid (Fig. 2E). Moreover, each centriole still retained localization of Unc-GFP at the DCC, indicating that the structure it labels remains intact in the presence of nocodazole.

#### The formation of an axoneme does not require centriole docking to the plasma membrane

**Fig. 3.**
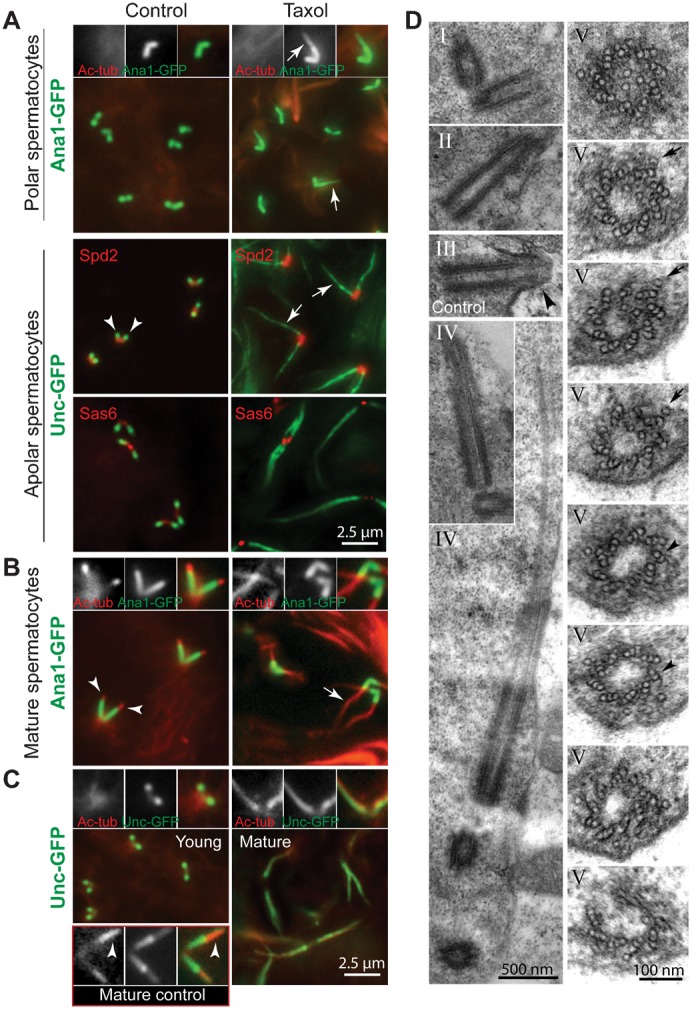
Microtubule stabilization leads to axoneme elongation. (A–C) Left panels are controls, right panels are Taxol-treated. (A) In control and Taxol-treated apolar spermatocytes D-SPD2, D-SAS6 and Ana1-GFP localization at centrioles is similar, whereas Unc-GFP localizes to a distinct focus at the distal tip of centrioles in control cells (arrowheads) and to elongated thin filaments (arrows) in Taxol-treated ones. Note that Ana1-GFP signal was elongated slightly in Taxol-treated young spermatocytes (arrow in inset). Ac-tub: acetylated tubulin. (B) In mature spermatocytes, staining for Ac-tub shows that the axonemes (arrowheads in control) are significantly elongated upon Taxol treatment (arrow). (C) Comparison of young and mature spermatocyte patterns of Unc-GFP and Ac-tub show the development of the Ac-tub cilium signal in mature axonemes, whereas these signals are both expanded along the elongated axonemes of Taxol-treated spermatocyte cilia. A control mature spermatocyte is shown in the bottom three inset panels, to show, by comparison, that Unc-GFP is less prominent in the cilium (arrowhead) compared to Taxol-treated early spermatocytes. (D) EM images of Taxol-treated spermatocytes. All images are Taxol-treated specimens except for (III). Centrioles in Taxol treated young primary spermatocytes (I) appear normal, whereas in treated apolar spermatocytes (II) they migrate at the surface as normal, but do not dock to the cell membrane, as do centrioles in control apolar spermatocytes (III), which form dome-like structures (arrowhead). Taxol treatment of polar spermatocytes induces microtubule extensions from the distal end of centrioles (IV). Serial cross sections, taken at 50 nm intervals, show that the proximal region of these extensions is composed of nine triplet microtubules, the mid region contains both triplet and doublet (arrowheads) microtubules, whereas the distal region has an incomplete wall (V); note the supernumerary tubules inside the lumen and outside running parallel to the axoneme (arrows). Scale bars: 2.5 µm (A,C), 500 nm (D) (IV), 100 nm (D) (V).

In polar (Fig. 1B) and young apolar (Fig. 3A) spermatocytes the two centriole pairs normally reside at the cell surface, and are marked distinctly as basal bodies by Unc-GFP. However, following treatment with 5 µM Taxol, polar (Fig. 1B) and young apolar (Fig. 3A) spermatocytes displayed an intricate elongated network of Unc-GFP-labelled filaments. These filamentous Unc-GFP-decorated structures were not seen in the cytoplasm of control cells.

To determine whether the Unc-GFP threads induced by Taxol treatment were elongated centrioles, ciliary axonemes, or both, we stained for proteins that differentially label these structures. We stained centrioles with antibodies against D-PLP and Cnn (not shown), which localize to the proximal region of centrioles, and against D-SPD2, D-SAS6, γ-tubulin (not shown), and Ana1-GFP, which localize along the entire length of centrioles (Fig. 3A,B). All six of these centriole proteins localized to centrioles at the base of the threads in Taxol-treated spermatocytes similarly to their localization at control centrioles (Fig. 3A,B and data not shown). Only Ana1-GFP showed a weak expansion of the signal at the distal end of the centrioles in early polar spermatocytes, at a stage that precedes cilium assembly, following Taxol treatment (Fig. 3A). This expansion of Ana1-GFP was not seen in later spermatocytes (Fig. 3B). None of these centriole/centrosome proteins were localized along the filaments decorated with Unc-GFP that extend from the end of the centrioles, suggesting that these elongated structures are not extended centrioles. On the other hand, staining with antibodies directed against acetylated tubulin, which recognizes stable axonemal microtubules in cilia and flagella, indicated that the Taxol-induced Unc-GFP-decorated filaments are elongated axonemes. Normally, spermatocyte axonemes are short and grow longer as spermatocytes mature into prophase (bottom of Fig. 3C), but do not grow as long as the axonemes that assemble in the presence of Taxol (Fig. 3C). Thus, the filaments that extend from centrioles in Taxol-treated spermatocytes appear to be elongated axonemes rather than extended centrioles.

These data show that Unc localization to the DCC is aberrant in Taxol-treated early spermatocytes, providing further evidence for this compartment's involvement in basal body docking to the membrane. Instead, Unc was localized uniformly along the long cytoplasmic axonemes that assemble following Taxol treatment. Together, these data indicate that the Unc-labelled structure at the distal centriole, the transition zone or perhaps a proximal substructure of the transition zone, fails to assemble and to function normally upon Taxol treatment, leading to membrane docking failure. Interestingly, this defect did not occur with nocodazole treatment. Instead, with nocodazole treatment, Unc remained focused at the DCC despite the loss of the cilium, and the centriole was still capable of docking to the membrane.

To more clearly define the elongated axonemes induced by Taxol at the ultrastructural level we examined spermatocyte centrioles and cilia by EM. Young primary spermatocytes displayed centriole pairs inside the cytoplasm that appeared normal following Taxol treatment (Fig. 3DI). They were found to have migrated toward the surface; however, they failed to dock with the plasma membrane (Fig. 3DII). This contrasts with control apolar spermatocytes, where the centrioles dock to the plasma membrane and a dome-like structure forms around the distal end of the centriole (Fig. 3DIII) (A. D. Tates, PhD thesis). Moreover, ultrathin serial sections of apolar Taxol-treated spermatocytes showed that the distal ends of the centrioles have extended axonemes that are significantly longer than those found in control cilia (Fig. 3DIV). These elongated axonemes are contained within the cytoplasm and do not protrude from the cell surface (Fig. 3DIV). The structural organization of the centriole microtubules appeared normal, except that the central singlet tubule, a dynamic singlet tubule characteristic of the spermatocyte centriole (A. D. Tates, PhD thesis; Riparbelli et al., 2012; Carvalho-Santos et al., 2012; Roque et al., 2012), was often present in two or three copies within the centriole lumen (Fig. 3DV). Curiously, the elongated axonemes induced by Taxol contain triplet microtubules, resembling centrioles in their architecture. The distal part of these elongated axonemes are more irregular, and have an incomplete radial wall, indicating that the axoneme grows unevenly along the proximo-distal axis with microtubule blades terminating at different distances from the proximal end seen in both the sagittal (Fig. 3DIV) and the cross sectional (Fig. 3DV) views.

The Taxol-induced filaments are therefore composed of a proximal centriole and an abnormal axoneme that elongates considerably within the cytoplasm. Unc is localized uniformly along the axoneme but does not accumulate at the junction between the centriole and the axoneme, suggesting that Taxol disrupts the DCC. Moreover, a ciliary membrane did not surround Taxol-induced axonemes as normally occurs with spermatocyte cilium assembly. Thus, Taxol blocks centriole docking to the plasma membrane, but amplifies axoneme growth in the cytoplasm by extending the triplet microtubule composition of the centriole rather than translating this into the typical doublet configuration that is normally found in ciliary axonemes.

One interpretation of these results is that Taxol perturbs the normal assembly of the DCC or the transition zone, disrupting the docking of the centriole to the plasma membrane, a prerequisite step for assembling a membrane-encased axoneme during ciliogenesis. Primary cilia normally contain a membranous sheath surrounding the axoneme and a transition zone structure at the distal tip of the basal body, which regulates trafficking into the cilium (Reiter et al., 2012; Szymanska and Johnson, 2012). Both these features are missing or disrupted in the centriole extensions found in spermatocytes treated with Taxol prior to membrane docking, but are present in cilia assembled prior to Taxol treatment (see below). These results indicate that Taxol disrupts the proper assembly of the transition zone, or some proximal component of it, but not its maintenance. The consequence of this disruption is failure of the basal body to dock to the membrane and it also alters the transition of centriolar triplet microtubules to doublets in the axoneme during its assembly. This scenario implicates the requirement of factors that destabilize centriolar microtubules at the distal end prior to docking at the membrane. A candidate for this activity is the kinesin-13 family of motor proteins (Delgehyr et al., 2012; Kobayashi et al., 2011; Piao et al., 2009).

Since Taxol blocked centriole docking to the membrane, we next sought to determine the effects of Taxol on ciliary axonemes after the docking step, when access to the cilium may be regulated as it is for primary cilia in other systems.

#### Microtubule stabilization leads to cilium extension

The primary spermatocyte stage lasts about 90 hours in Drosophila melanogaster (Lindsley and Tokuyasu, 1980). It has been estimated that about 5 hours elapses from the time that primary spermatocytes enter the first meiosis to the onset of spermatid differentiation (Cenci et al., 1994). Since we treated testes with Taxol for 24 hours, our specimens contain various stages of spermatogenesis allowing us to evaluate whether the treatment impacted centriole migration, centriole docking, cilium formation and also later stages of spermiogenesis. Our above findings indicate that centriole migration was not perturbed by Taxol, but docking to the membrane was. Next we looked at the effect of Taxol on ciliary axonemes formed from centrioles that had completed the docking stage.

**Fig. 4.**
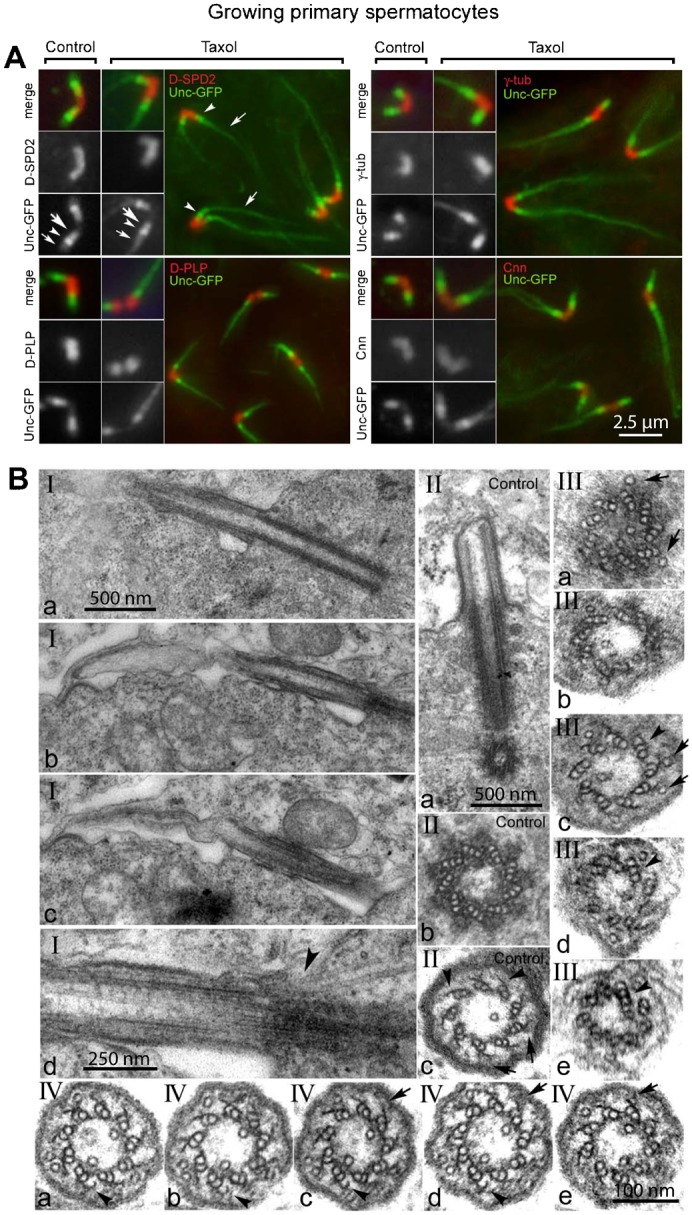
Microtubule stabilization leads to cilia of extended length. (A) Unc-GFP (green) is localized in three distinct regions in both control and treated growing primary spermatocytes: along the centriole (large arrows) that partially or totally overlaps D-PLP, D-SPD2, γ-tubulin or Cnn centriole markers (red), a prominent localization at the distal centriole (arrowheads), and distal localization to the axoneme (small arrows). The axoneme localization is elongated in Taxol treated spermatocytes. (B) Ultrastructure of cilia in Taxol treated (I,III,IV) and control (II) growing primary spermatocytes. After microtubule stabilization with Taxol the primary cilia increased in length (I) compared with controls (II), but the distal axoneme is disorganized with disruption or loss of the radial blades of microtubule doublets. A magnified view of the transition region between the centriole and the ciliary axoneme shows ectopic microtubules that extend from the cytoplasm into the cilium in Taxol-treated spermatocytes (Id, arrowhead). Cross sections of the control centriole show nine triplet microtubules that transform into nine doublets in the ciliary axoneme: note that lateral projections (IIc, arrowheads) protrude from the B tubules toward a dense material near the ciliary membrane (IIc, arrows). (III) Serial cross sections at 50 nm intervals show that the centriole in Taxol-treated spermatocytes is formed by triplet microtubules in the usual nine-fold symmetry; however, more than the usual single tubule are found within the lumen of the centriole (IIIa) and ectopic microtubules run parallel to the centriole and the axoneme (arrows in IIIa,c). Although most of the triplets lost their C-tubule, some of them retain this along the length of the axoneme (arrowheads), which has an incomplete wall in the distal section (IIIe). (IV) Serial 50 nm cross sections of the mid region of a ciliary axoneme show incomplete loss of the C-tubule that appears as a hook-like projection from the B-tubule (arrowheads). The projections emerging from the B-tubule contact clusters of electron-dense material below the plasma membrane (arrows), as in controls. Scale bars: 2.5 µm (A), 500 nm (B) (Ia, IIa), 250 nm (B) (Id), 100 nm (B) (IVe).

Treatment of late spermatocytes with Taxol, after the centrioles had docked at the membrane, also resulted in elongated axonemes decorated with Unc-GFP (Fig. 4A). The centrioles, identified with staining for D-SPD2, D-PLP, γ-tubulin or Cnn, again appeared unaffected morphologically by Taxol treatment (Fig. 4A).

EM analysis confirmed that, despite their significant length, the Taxol-induced GFP-Unc-labelled filaments represent cilia of extended length. Elongated cilia that emerge from the plasma membrane from docked centrioles appear in Taxol-treated growing primary spermatocytes (Fig. 4BI). Serial sagittal sections showed that the proximal part of these structures have an apparently normal organization compared to untreated controls (compare Fig. 4BI and Fig. 4BII), whereas the distal region of the cilium was disorganized and composed of “wavy” microtubules (Fig. 4BIb,c). Inspection of the transition region between the centriole and the ciliary axoneme revealed longitudinal microtubules close to the centriole wall that appeared to extend from the cytoplasm into the cilium through the transition zone (Fig. 4BId,III,IV).

Typically, a singlet tubule runs along the lumen of the centriole and is contiguous into the ciliary axoneme (Fig. 4BII). The centrioles of treated cells still retained the nine-fold symmetry, but one or two additional tubules were often found inside their lumen (Fig. 4BIIIa). Another feature of Taxol-treated spermatocyte cilia that is not normally seen are single microtubules that run parallel to the centriole wall outside of the axoneme (Fig. 4BIIIa,c), which may correspond to the microtubules seen in sagittal sections (Fig. 4BId). Cross sections revealed these supernumerary microtubules inside and outside of the axoneme (Fig. 4BIII,IV).

The structure of the ciliary axoneme appeared normal along most of its length, containing nine doublet microtubules (Fig. 4BIII,IV). There was the occasional retention of triplet microtubules (Fig. 4BIII). This contrasts with the axonemes that grew in the cytoplasm from undocked centrioles under the influence of Taxol, which had triplet microtubules along much of its length (Fig. 3BV). However, the structure of these elongated axonemes deteriorated at their distal ends, and the numbers of doublets decreased toward the end of the cilium (Fig. 4BIII). Lateral projections emerge from the B-tubule (Fig. 4BIV), as observed in controls (Fig. 4BIIc). One or two C-tubules persisted along the proximal end or appeared as incomplete tubules with the appearance of hook-like projections from the B-tubule (Fig. 4BIV).

These findings show that, remarkably, microtubule stabilization after centriole docking to the plasma membrane leads to the formation of longer axonemes. This contrasts with findings in vertebrate cells in culture, where stabilization of microtubules with Taxol, which reduces the free tubulin pool available, resulted in loss of cilia (Sharma et al., 2011). The Taxol-induced elongated cilia we show here resemble normal cilia in several respects, with axonemes composed mostly of microtubule doublets enveloped by the plasma membrane and with distinct projections that link the axonemal doublets to the ciliary membrane. These results suggest that centriole docking at the plasma membrane is a prerequisite to build a cilium, although this step is not necessary to assemble an axoneme. Thus, the Taxol experiments we describe here indicate that an axoneme can assemble from a basal body without the proper regulation by the transition zone.

These Taxol-induced extensions resemble axonemes as they do not recruit centriolar proteins and are modified by acetylated tubulin. Interestingly, the microtubule blades, when viewed by EM, have the architecture of centrioles with a triplet composition, although often they are of mixed composition with doublets. This feature might suggest that axonemes elongate as triplets, but that some destabilizing activity normally removes only the C-tubule in the transition zone, which in our experimental conditions is disrupted by Taxol stabilization.

### Taxol does not affect axoneme growth in mature spermatocytes

**Fig. 5.**
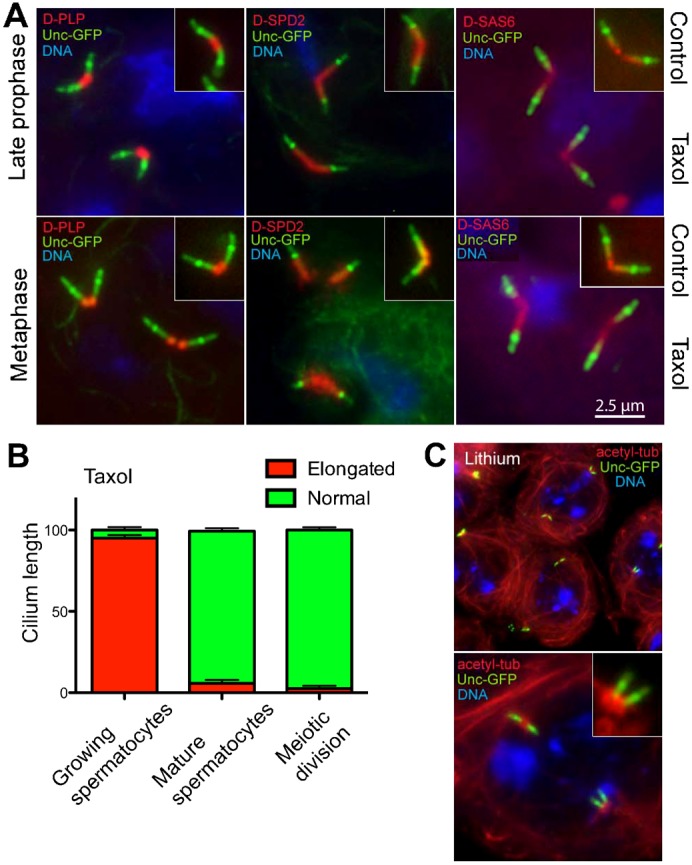
Taxol does not affect cilium length in mature spermatocytes. (A) The length of the cilia in Taxol-treated cells, labeled by Unc-GFP staining (green), is comparable to that observed in control cells. Co-staining of D-PLP, D-SPD2 and D-SAS6 (red) to show the centrioles. (B) Quantification of axoneme elongation scored in the indicated cell types based upon the presence of elongated Unc-GFP filaments at the distal end of the centriole. Error bars represent SEM. (C) Treatment with lithium leaves spermatocyte cilia intact. Shown are two examples of spermatocytes treated with 20 mM LiCl for 24 hrs. Scale bar: 2.5 µm (A).

Spermatocyte cilia normally grow to their maximum length at the end of the first prophase and then persist through both meiotic divisions, until the spermatid stage. The cilia at the spindle poles in Taxol treated mature spermatocytes had the same Unc-GFP pattern as the control (Fig. 5A). Thus, Taxol treatment had no overt effect on the architecture of these fully assembled cilia in mature primary spermatocytes during the meiotic divisions (Fig. 5B).

These data show that Taxol promotes assembly of longer axonemes or cilia when they are growing, but has no apparent effect on mature spermatocytes and during the meiotic divisions once the centrioles and cilia have matured to their full length. These results indicate that cilia formation and the control of their length are regulated differently during spermatocyte growth. This suggests that a barrier to Taxol may have been imposed by this late stage. This might be due to changes in transition zone functionality. On the other hand, there might be changes to the axonemal tubulins or the transport of tubulin that render these mature axonemes less sensitive to Taxol stabilization.

Altogether these findings show that Drosophila spermatocyte primary cilia exhibit several unique characteristics. One possibility that has been proposed is that these are not mature cilia, but rather an unusually long transition zone structure (Enjolras et al., 2012; Riparbelli et al., 2012). The lateral projections that decorate the whole length of the axoneme support this latter interpretation. However, if these structures are transition zones, then they represent extraordinarily long ones. The transition fibers found in vertebrate cells are Y-shaped radial structures that emerge between the A and B tubules and directly link the basal body to the ciliary membrane. In contrast, the lateral links along the Drosophila ciliary axoneme extend obliquely from the B-tubule, appearing as remnants of the C-tubule, and do not reach the ciliary membrane but contact aggregates of dense material below the membrane. Thus the lateral links found along the axoneme of Drosophila spermatocytes do not resemble the canonical transition fibers found in other systems where they form a selective barrier between the ciliary compartment and the cytoplasm (Ishikawa and Marshall, 2011).

### GSK-3 inhibition has no observable impact on spermatocyte cilia

We then asked if alternative perturbations of the microtubule cytoskeleton would influence the assembly dynamics of spermatocyte primary cilia. We examined the effect of blocking the conserved multifunctional kinase glycogen synthase kinase-3 (GSK-3), which was shown to influence the integrity of the cytoplasmic network of microtubules (Izumi et al., 2008). To inhibit GSK-3 we incubated Unc-GFP expressing testes with 20 mM lithium chloride, an established inhibitor of GSK-3 (O'Brien and Klein, 2009). Although the microtubule cytoskeleton network was altered by lithium treatment, neither centrioles nor cilia were overtly affected by it at any stage (Fig. 5C, mature spermatocytes). Inhibition of Gsk3 kinase by lithium chloride resulted in elongated flagella in Chlamydomonas (Wilson and Lefebvre, 2004; Nakamura et al., 1987) and overly long primary cilia in mammalian cells (Ou et al., 2009; Miyoshi et al., 2009), but did not affect the length and shape of cilia in Drosophila spermatocytes. This result further exemplifies that in Drosophila spermatocytes the modulation of the ciliary length does not appear to require signalling that is used in systems that require IFT for axonemal morphogenesis.

## Conclusion

Here we show that the primary cilia found in Drosophila spermatocytes are uniquely sensitive to drugs that affect microtubule stability or dynamics. We show that spermatocyte cilia and spermatid flagella are sensitive to nocodazole, a microtubule-destabilizing drug. Nocodazole did not block centriole duplication or migration and docking to the plasma membrane, but did block axoneme assembly or maintenance. On the other hand Taxol, a microtubule-stabilizing drug, caused spermatocyte axonemes to grow unusually long pre- or post-docking to the plasma membrane, but in fully mature cilia it did not affect axoneme length. Together, these data show that spermatocyte cilia are divergent from typical mammalian cilia and might represent a separate class of cilia that operate in an IFT-independent manner.

In addition to the above ultrastructural differences, Drosophila spermatocyte cilia diverge from conventional primary cilia by several additional aspects. The axonemes of vertebrate cell primary cilia elongate from the distal end of the basal body, the mature mother centriole, which does not change in length during this process. By contrast, both mother and daughter centrioles assemble cilia in Drosophila spermatocytes, and they do so in G2 phase rather than in G1 or G0 cells. Moreover, centrioles and ciliary axonemes elongate simultaneously in primary spermatocytes. How the centrioles are able to elongate while at the same time template the assembly of the axoneme is unknown, but suggests some novel dynamics at the distal end of these centrioles. Altogether, there are several unique properties of spermatocyte cilia that distinguish them from their counterparts in vertebrate cells; one or more of these may explain the differences in Taxol and nocodazole sensitivities that we report here.

## Materials and Methods

### Drosophila strains

The stock containing the Unc-GFP transgene was described previously (Baker et al., 2004). The Ana1-GFP was described previously (Blachon et al., 2009). Flies were raised on standard Drosophila medium at 24°C.

### Antibodies and reagents

Chicken anti-DSAS-6 (1:1.000), rabbit anti-DSPD-2 (1:500), and chicken anti-DPLP (1:1.500) were described previously (Rodrigues-Martins et al., 2007). The rabbit anti-Cnn (1:400) was kindly provided by Eyal Schejter (Vaizel-Ohayon and Schejter, 1999). Mouse anti-**γ**-tubulin-GTU88 (1:100) and mouse anti-acetylated tubulin (1:100) were from Sigma–Aldrich. Alexa Fluor 488- and 555-secondary antibodies (1:800) were purchased from Invitrogen. Paclitaxel (Taxol, from Taxus brevifolia), nocodazole, Dimethyl sulfoxide (DMSO), LiCl, and Shields and Sang M3 Insect Medium were purchased from Sigma. Taxol, nocodazole and LiCl were dissolved at 1 mg/ml in DMSO and stored frozen at −20°C.

### Culture and drug treatment experiments

Testes were dissected from pupae between 5–7 days after pupation. At this stage testis contain cysts at the spermatogonial, spermatocyte, and spermatid stages. Testes were then transferred in a 200 µl of M3 medium into a sterile 24-well plate. To assess the effect of microtubule depolymerization on cilia length the dissected testes were cultured at 24°C for 24 hours in M3 medium containing 100 nM or 10 µM nocodazole. To analyze the effect of microtubule stabilization testes were incubated 24 hours in M3 medium containing Taxol 5 µM. Recovery from nocodazole 10 µM was made by a further 12 hour incubation of the treated testes in nocodazole-free M3 medium. To examine the effect of GSK-3 on cilia dynamics we incubated dissected testes in M3 medium containing 20 mM of LiCl. Controls testes were incubated in M3 medium containing the vehicle (DMSO). The stages affected by the indicated treatments were inferred based on the 24 hr lapse time and the reported times for progression through the different stages (Fig. 1A). Specimens were fixed and stained following 24 hr drug or vehicle treatments, so no long-term effects were evaluated. These experiments were repeated at least ten times.

### Indirect immunofluorescence staining

Testes were washed in M3 medium for 10 minutes and then in phosphate buffered saline (PBS) for 5 minutes. Then testes were placed in a small drop of 5% glycerol in PBS on a glass slide and squashed under a small cover glass and frozen in liquid nitrogen. After removal of the coverslip the samples adhered to the slides were immersed in methanol for 10 min at −20°C. For localization of microtubules and centriole/centrosomal components, testes were washed for 15 min in PBS and incubated for 1 h in PBS containing 0.1% bovine serum albumin (PBS–BSA) to block non-specific staining. Testes were incubated overnight at 4°C with the specific antisera against Cnn, D-SPD2, D-PLP, and DSAS-6 and then with anti-acetylated tubulin antibody for 4–5 h at room temperature. For localization of γ-tubulin testes were incubated in the GTU88 antibody. After washing in PBS–BSA the samples were incubated for 1 h at room temperature with the appropriate secondary antibodies. In all cases DNA was visualized with incubation of 3–4 min in Hoechst. Testes were mounted in small drops of 90% glycerol in PBS.

Images were taken by using an Axio Imager Z1 (Carl Zeiss) microscope equipped with an AxioCam HR cooled charge-coupled camera (Carl Zeiss). Gray-scale digital images were collected separately and then pseudocolored and merged using Adobe Photoshop 7.0 software (Adobe Systems).

### Transmission electron microscopy

Testes incubated in M3 medium in the presence or absence of drugs were washed twice in the free-drug medium, then 5 minutes with PBS and finally fixed overnight at 4°C in 2.5% glutaraldehyde in PBS. After washing for 30 min in PBS the material was post-fixed in 1% osmium tetroxide in PBS for 2 h. After washing for 15 minutes in distilled water the samples were dehydrated in a graded series of alcohols, and then embedded in an Epon–Araldite mixture and polymerized at 60°C for 48 h. Ultrathin sections obtained with an LKB ultratome Nova were stained with uranyl acetate and lead citrate. Observations were performed with a Tecnai Spirit Transmission Electron Microscope (FEI) operating at 100 kV equipped with a Morada CCD camera (Olympus).

### Statistics

Statistical analysis of centriole numbers utilized Prism software, where cells in individual cysts were counted as separate groups (counts within each cyst were tallied as *n* = 1 experiment). The error was measured as standard error of the means (SEM). Significance was measured using the two-tailed unpaired Student's t test. This was not measured for the mature spermatocytes because the numbers of centrioles did not vary in the control group among 18 cysts (every cell counted had 4 centrioles). For significance ranking values, *p<0.05, **p<0.01, ***p<0.001.
